# A New Prediction Method for the Ultimate Tensile Strength of Steel Alloys with Small Punch Test

**DOI:** 10.3390/ma11091491

**Published:** 2018-08-21

**Authors:** Jose Calaf Chica, Pedro Miguel Bravo Díez, Mónica Preciado Calzada

**Affiliations:** Department of Civil Engineering, University of Burgos, Avenida Cantabria s/n, 09007 Burgos, Spain; pmbravo@ubu.es (P.M.B.D.); mpreciado@ubu.es (M.P.C.)

**Keywords:** Small Punch Test, SPT, ultimate tensile strength, strain-hardening

## Abstract

The load–deflection curve acquired from the Small Punch Test (SPT) is used to obtain the mechanical properties of materials using different correlation methods. The scattering level of these regressions tends to be high when a wide set of materials is analyzed. In this study, a correlation method based on a specific slope of the SPT curve was proposed to reduce scattering. Assuming the Ramberg–Osgood hardening law, the dependence of the SPT curve slope on the yield strength and the hardening coefficient is demonstrated by numerical simulations (FEM). Considering that the ultimate tensile strength could be obtained from the hardening coefficient, a response surface of the ultimate tensile strength with the yield strength and SPT curve slope, along with its equation, is presented for steel alloys. A summary of steel mechanical properties, based on the Boiler and Pressure Vessel Code (BPVC) and limited to yield strengths lower than 1300 MPa, is shown to select a set of experimental tests (tensile tests and SPTs) for which the range is completely covered. This experimental analysis validates the previous FEM analyses and the validity of the proposed correlation method, which shows more accurate correlations compared to the current methods.

## 1. Introduction

The Small Punch Test (SPT) was initially developed in the 1980s as a cost-effective method to test post-irradiated materials [1,2]. The applicability of this test has been extended to other applications where miniaturization has made it possible to estimate the mechanical properties of small amounts of materials. Widespread and uninterrupted research using the SPT has led to a complete family of methods to characterize mechanical properties such as Young’s modulus, the yield strength and ultimate tensile strength [3,4,5], a ductile–brittle transition [6], fracture properties [7,8,9,10], etc. The development of a CEN Code of Practice [11] and the effort invested to publish an European standard in 2019 [12] for the application and use of the Small Punch Test method for metallic materials shows the aim to standardize the SPTs performed by each lab, and will lead to the comparability of research in different places and sectors.

**Figure 1:**
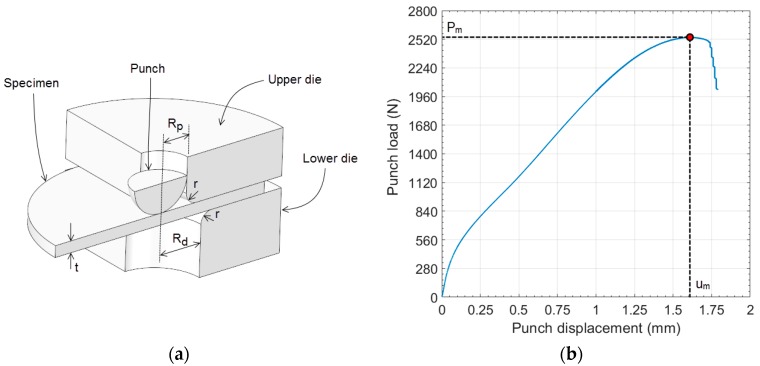
Small Punch Test (SPT) setup (**a**) and experimental load–displacement curve (**b**).

Figure 1 shows a schematic representation of the SPT. A specimen with a thickness of 0.5 mm and a diameter ≥8.0 mm is clamped between two dies and punched until failure with a spherical punch. The main geometry data of the SPT are: *R_p_* = 1.25 mm, *R_d_* = 2.0 mm, and *r* = 0.5 mm.

The resulting data recorded during the SPT are the load/displacement curves (see Figure 1), from which different data are extracted. For the ultimate tensile stress *σ_u_*, the most widely used method for the estimation of this mechanical property is the maximum load *P_m_* of the SPT curve (see Figure 1). Mao and Takahashi [7] developed the method for estimating *σ_u_* with Equation (1):

(1) $$\;\sigma_{u}=\beta_{1}\cdot \frac{P_{m}\;}{t^{2}}+\beta_{2}$$

where *β*_1_ and *β*_2_ are the correlation coefficients, *P_m_* is the maximum load of the SPT curve, and *t* is the specimen thickness. 

Garcia et al. [13] recommended the use of *P_m_/(t·u_m_)* instead of *P_m_/t*^2^ (see Figure 1 for *u_m_* calculation), and other researchers developed alternatives that have the use of the maximum load *P_m_* of the SPT curve [14] in common. Kumar et al. [15] showed in their research that the SPT specimen, in the proximity of the maximum load *P_m_*, had initial cracks. Thus, material damage is initiated before reaching the maximum load of the SPT curve. The ultimate tensile strength is obtained from the uniaxial tensile test when damage or cracks have yet to be initiated. Thus, the maximum load *P_m_* does not seem to be a good parameter to be correlated with *σ_u_*, because it is influenced by the damage properties of the material. This was pointed out by Campitelli [16] using a finite element analysis, and concluded that the stress–strain state of the SPT specimen at maximum load *P_m_* is completely different from that at necking in a standard tensile test.

Based on these previous considerations, Altstadt et al. [17] proposed an alternative parameter obtained from the SPT curve: the *F_i_* load. This data was the force at a fixed punch displacement of 0.645 mm. This method was based on the shape of the SPT curves obtained by the finite element analyses of hypothetical materials. When the elastic mechanical properties, yield strength, and ultimate tensile strength were fixed, and the strain for this ultimate tensile strength was modified, SPT curves showed pseudo-intersections at a punch displacement of around 0.645 mm. For other fixed ultimate tensile strengths, the pseudo-intersections were found in a punch displacement close to the same displacement of 0.645 mm. This was investigated for hypothetical materials with an elastic modulus of *E* = 212,000 MPa and an initial flow stress of *σ_y_*_0_ = 570 MPa. Different elastic modulus or initial flow stresses would give different punch displacements for the pseudo-intersections. An experimental comparison between the widely used method of *P_m_* correlation (*F_m_* in their paper) with the ultimate tensile strength and their new *F_i_* method was performed with the coefficient of determination *R^2^* of both correlations:

(2) $$\;R^{2}\left\{F_{i}\;\right\}=0.870$$

(3) $$\;R^{2}\left\{P_{m}\;\right\}=0.747$$

where the *F_i_* method showed lower deviations than the *P_m_* method.

The present research was initiated taking into consideration the same fact exposed previously by Kumar et al. [15] and followed by Altstadt et al.: points prior to the *P_m_* are more suitable for the estimation of ultimate tensile strength *σ_u_*.

This article is focused on steel alloys and the obtaining of a new and more reliable correlation method for the ultimate tensile strength. The following steps were taken:

(a) Summary of steel mechanical properties. The aim of this review was to obtain a delimited surface of hardening coefficient versus yield strength for steel alloys. A specific selection of steels was considered to cover this entire surface for the experimental tests, with a maximum limit of 1300 MPa in the yield strength.
(b) SPT behavior analysis. A preliminary FEM analysis was performed to understand the behavior of the SPT specimen during the test. Based on this, a new correlation method for the ultimate tensile strength of the material was proposed.
(c) Hypothetical material analysis. A second FEM analysis was performed to obtain the correlation equations for the proposed method in the analysis indicated in the previous point (b).
(d) Finally, an experimental analysis was performed to verify the numerical study previously seen and the proposed correlation method. This experimental analysis covered a wide range of steel alloy properties with yield strengths lower than 1300 MPa.

## 2. Materials and Methods

The aim of this research was to obtain a correlation method for finding the ultimate tensile strength with the SPT in steel alloys. This investigation was experimentally limited to yield strengths lower than 1300 MPa.

**Figure 2:**
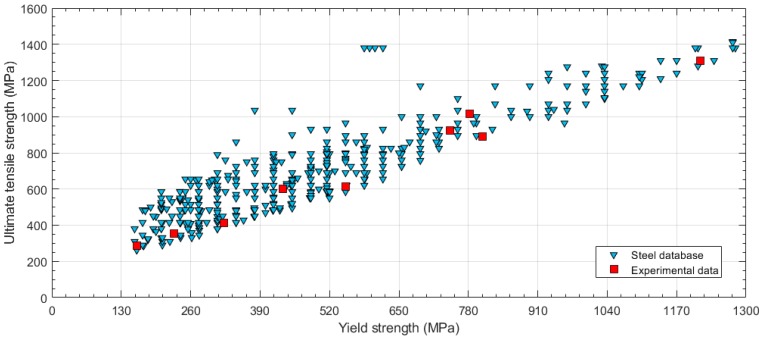
Steel database obtained from the ASME Boiler and Pressure Vessel Code (BPVC)-IIA and experimental data selection (ultimate tensile strength vs. yield strength).

In order to define the range of applicability of the correlation equation, the ample steel data collection published by ASME in its Boiler and Pressure Vessel Code (BPVC) [18] was used. Figure 2 shows the ultimate tensile strength versus the yield strength for steels contained in the BPVC-IIA-2017. The experimental steels used in this research are marked as red squares in the same graph. The objective was to include the experimental tests throughout the range of the surface covered by the steel database. 

In addition, an estimation of the hardening coefficients of the same database was done. A Ramberg–Osgood law [19] was used in this analysis following Equations (4) and (5).

(4) $$\;\varepsilon =\frac{\sigma }{E}+\varepsilon_{offset\;}{\left(\frac{\sigma }{\sigma_{y}}\right)}^{n}$$

(5)  n=ln(εm−σm Eεoffset)ln(σmσy)

where *ε_offset_* = 0.002.

Kamaya developed a method to estimate the Ramberg–Osgood law coefficients using yield and ultimate tensile strengths of the material [20]. For *ε_offset_* = 0.002, the relation obtained by Kamaya was:

(6) $$\;n=3.93{\left\{ln\left(\frac{\sigma_{u}\;}{\sigma_{y}}\right)\right\}}^{-0.754}$$

**Figure 3:**
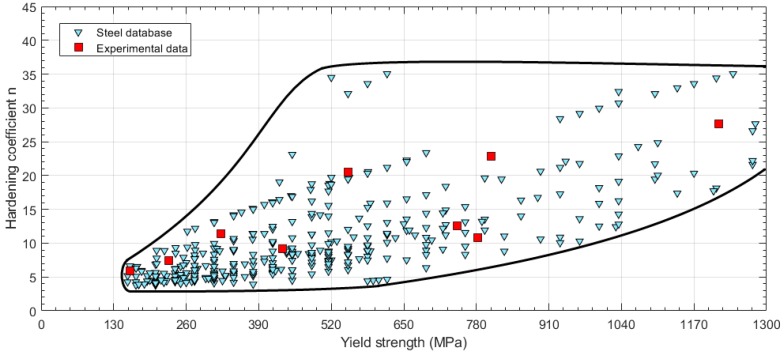
Steel database obtained from ASME BPVC-IIA and experimental data selection (hardening coefficient *n* vs. yield strength).

Once the strain hardening coefficient *n* of the material was obtained from Equation (6), the previous steel database presented in Figure 2 was changed to show the relationship between the hardening coefficient *n* and the yield strength (see Figure 3). A boundary limit for the steel database is represented in Figure 3. The hardening coefficient *n* was limited to a minimum value between *n* = 3 and *n* = 4, and the upper limit was set to levels of *n* = 37. The lower limit of *n* increases with the yield strength, and the same behavior is shown for the upper limit.

The selected experimental steels did not cover the higher values of *n*. High values of the *n* coefficient corresponded to materials with low strain hardening, so deviations in these high *n* values generate no significant changes in the strain-hardening curve.

For the “SPT behavior analysis”, a FEM simulation of the SPT was performed with a simplified linear hardening model to make the search for correlation easier. The specific material properties of this hypothetical material were: *E* = 200,000 MPa, *ν* = 0.3, *σ_y_* = 400 MPa, *E_p_* = 413 MPa, where *E_p_* is the plastic tangent modulus.

For the “Hypothetical material analysis”, 30 hypothetical materials were analyzed with an FEM simulation of the SPT. The hardening behavior applied in this analysis followed the Ramberg–Osgood Equations (4) and (5) with an isotropic hardening model.

The mechanical properties assigned for these hypothetical materials were fixed for their elastic behavior (*E* = 200,000 MPa and *ν* = 0.3), and were established as shown in Table 1 for their plastic behavior.

The SPT dimensions used in this numerical analysis were: *R_d_* = 2.0 mm, *R_p_* = 1.25 mm, *r* = 0.5 mm, and *t* = 0.5 mm (see Figure 1).

Nine steel alloys were used in the experimental procedure, and their mechanical properties were obtained with tensile tests following ASTM E8M [21] (see Table 2). Some of these experimental data were obtained in previous research [22].

## 3. Results

### 3.1. Numerical Analyses

**Figure 4:**
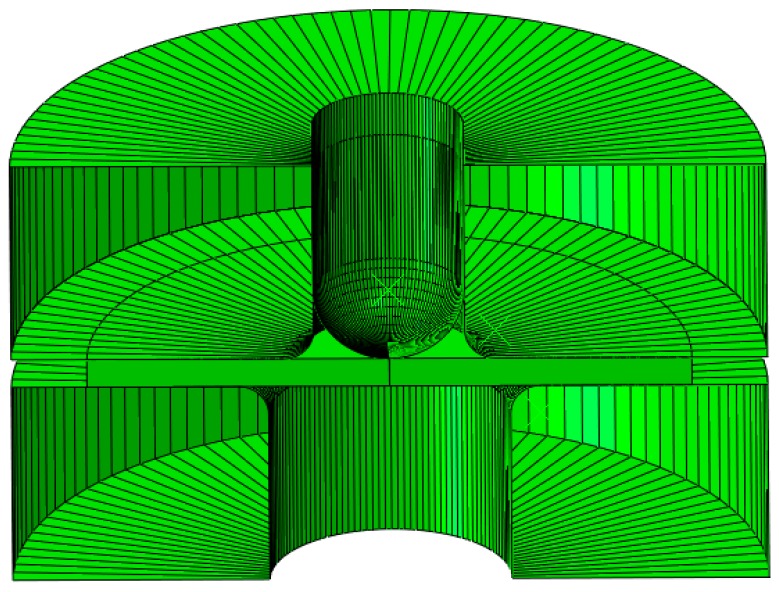
FEM set up of the SPT.

The SPT simulations (Figure 4 shows a view of the numerical model) were done in a similar way as used in previous research [22]. The mechanical properties used in these analyses are shown in Table 1.

#### 3.1.1. SPT Behavior Analysis

An FEM analysis was performed to understand the behavior of the SPT specimen during the test and the contribution of the material properties with respect to this behavior.

**Figure 5:**
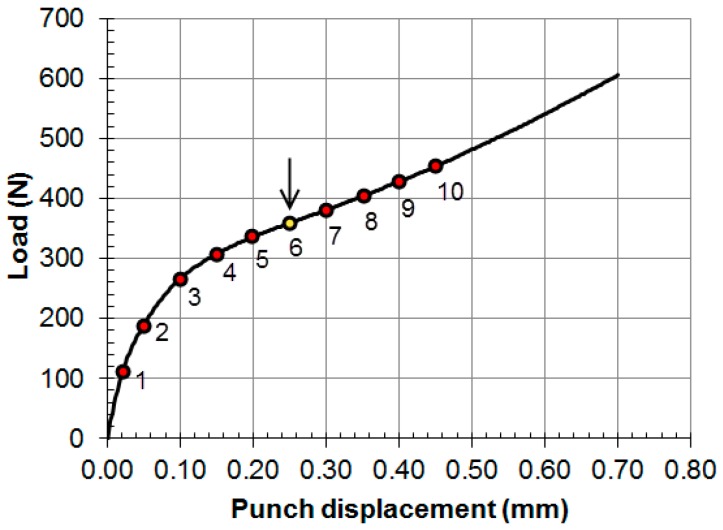
SPT curve for hypothetical bilinear material.

**Figure 6:**
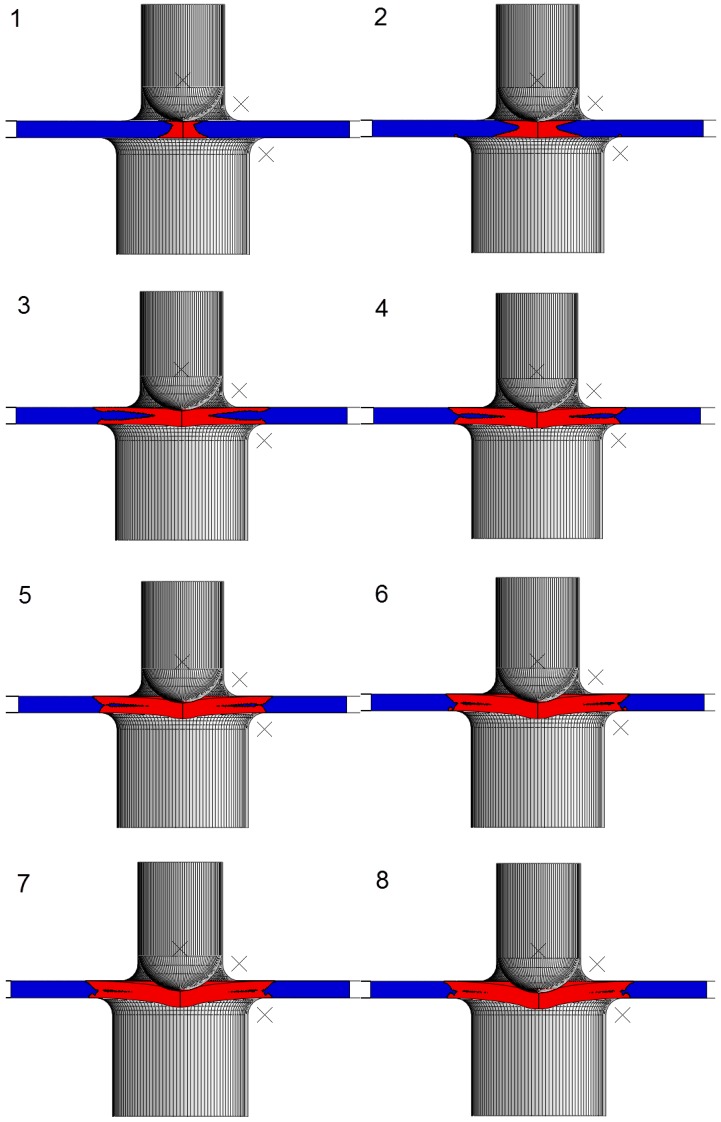
Elastic (blue) and plastic (red) zones in the SPT specimen at positions 1 to 8.

**Figure 7:**
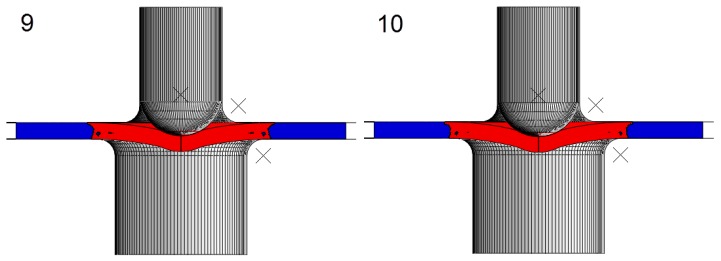
Elastic (blue) and plastic (red) zones in the SPT specimen at positions 9 to 10.

Figure 5 shows the SPT curve for the bilinear hypothetical material described in the previous section. Some punch displacements are indicated in this figure, and point 6 shows the punch displacement where the minimum slope of the SPT curve is located. Figure 6 and Figure 7 show bicolor images of the specimen where red is the material in the plastic regime, and blue is the material in the elastic regime. The volume of the specimen under the plastic regime grew until point 6, where the majority of the specimen that was not gripped between the dies was in the plastic regime.

The simplified material model used in this study guaranteed two rigidities: elastic stiffness controlled by the elastic modulus *E*, and plastic stiffness controlled by the plastic tangent modulus *E_p_*. The SPT curve is a load versus displacement curve that shows the global stiffness of the specimen in its slope. This global stiffness is a combination of two factors: the geometry of the test and the material stiffness distribution. The geometry of the test changes during the test because of changes in the contact surface area between the punch and the upper surface of the specimen. At greater punch displacements, the contact area is greater and the dependence on the geometry of the SPT’s stiffness increases. The material stiffness is obtained as a combination of two rigidities: the elastic stiffness and the plastic stiffness. An increase in the volume percentage of the specimen affected by plastic strain generates a decrease in the global specimen stiffness.

Thus, there are two factors for the changes in the slope of the SPT curve in the analyzed zones:

1. Non-linear contact stiffness originated by geometry changes. This rigidity grows during the test.
2. Material stiffness contribution. This rigidity diminishes during the test due to the growth of the yielded volume of the specimen (*E_p_* dominates over *E*). This diminishing in the rigidity reaches the minimum value when the entire specimen outside the gripped zone is yielded.

When the entire specimen is yielded, changes in the non-linear contact stiffness dominate the SPT curve slope. The slope reaches a minimum and begins to grow. This inflection point is shown in Figure 5 at point 6.

#### 3.1.2. Hypothetical Material Analysis

**Figure 8:**
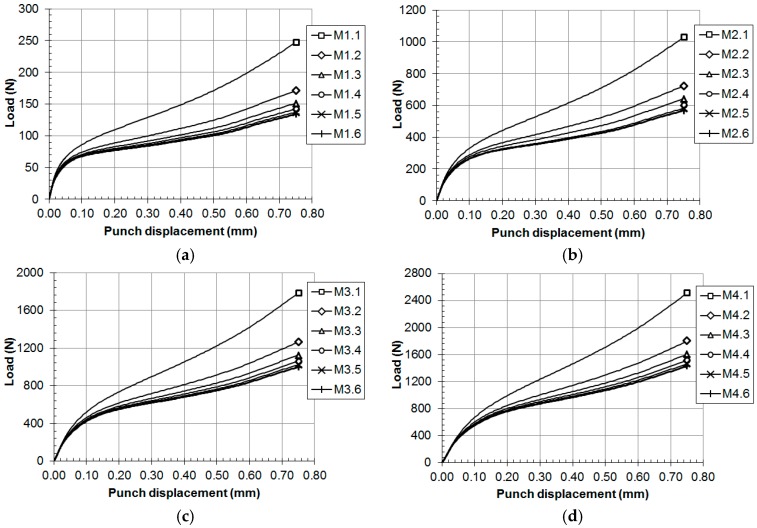
SPT curves for the hypothetical materials: (**a**) M1.x, (**b**) M2.x, (**c**) M3.x and (**d**) M4.x.

**Figure 9:**
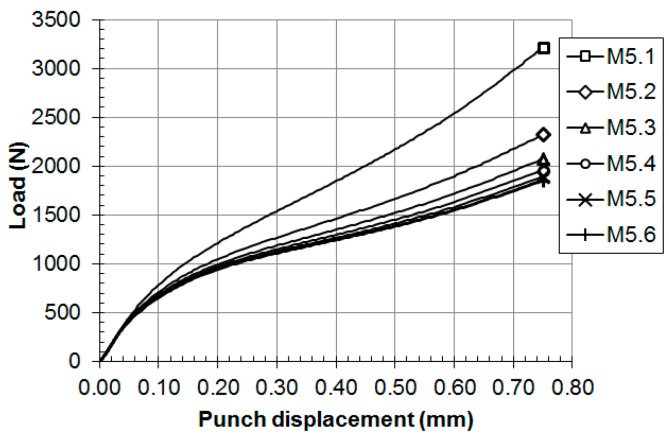
SPT curves for the hypothetical materials: M5.x.

Thirty hypothetical materials, M1.1 to M5.6 (see Table 1 for the mechanical properties of these materials), were simulated with the finite element model used in the previous section. Figure 8 and Figure 9 show the load versus displacement SPT curves for these hypothetical materials.

**Figure 10:**
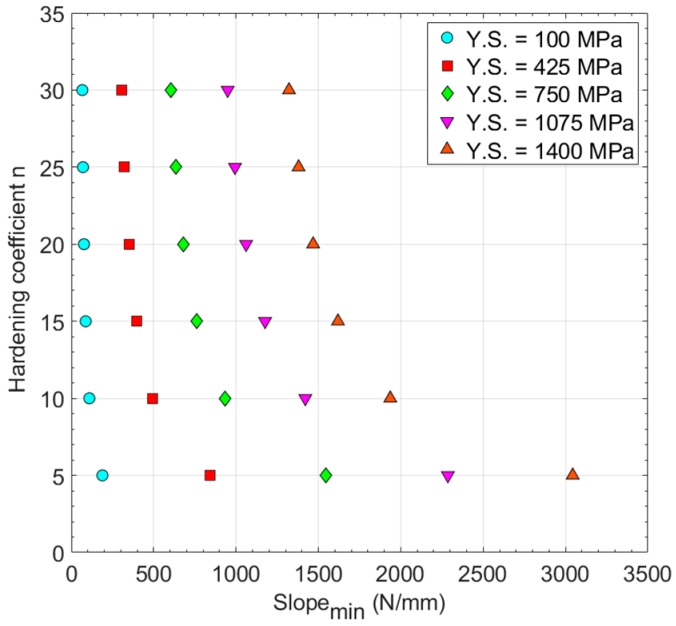
Hardening coefficient *n* vs. *Slope_min_* (Y.S.: yield strength).

The *Slope_min_* for all of the hypothetical materials is shown in Table 3 and Figure 10, where a dependency of the *Slope_min_* with both the hardening factor *n* and the yield strength is shown. For each value of yield strength, a correlation following Equation (7) seems to show a good fit. To determine the functions *φ*_1_ and *φ*_2_ of this equation, the Matlab^®^ Curve Fitting Tool^TM^ [23] was used with the non-linear least squares method.

(7) $$\;n=\frac{\varphi_{1}\left(\sigma_{y}\;\right)}{Slope_{min}-\varphi_{2}\left(\sigma_{y}\right)}$$

**Figure 11:**
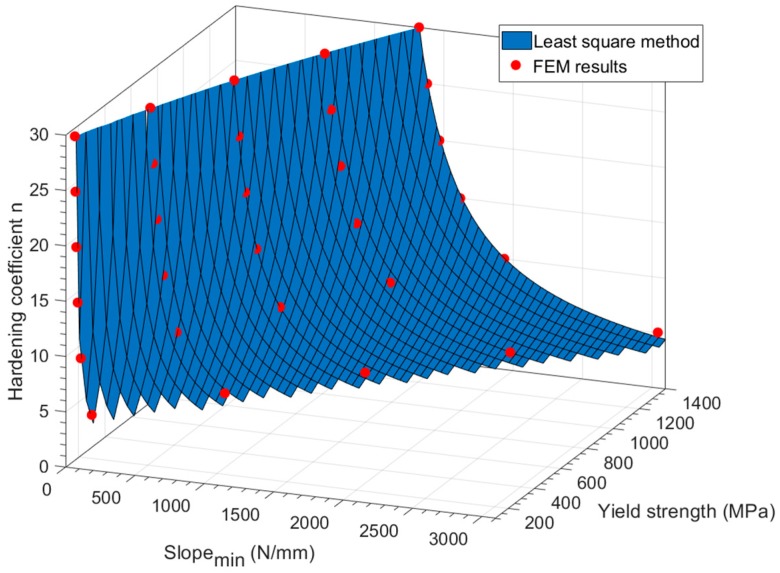
Correlation obtained from Matlab^®^.

Figure 11 and Equation (8) show the results obtained from the Curve Fitting Tool^TM^. The coefficient of determination of this regression was *R*^2^ = 0.993.

(8) $$\;n=\frac{6.645\sigma_{y}\;}{Slope_{min}-\left(2.222\times 10^{-4}\sigma_{y}^{2}+0.412\sigma_{y}\right)}$$

**Figure 12:**
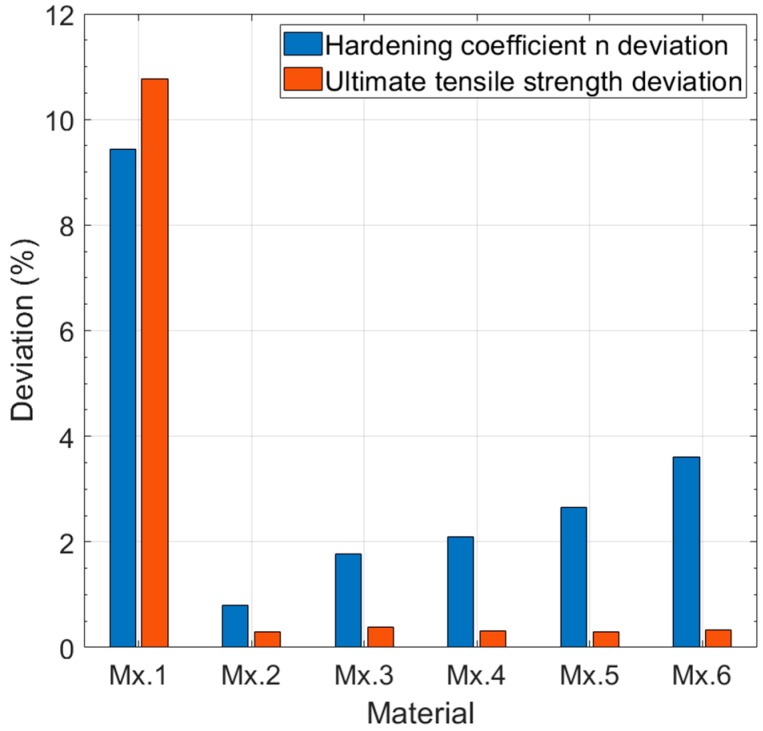
Comparison of the deviations of *n_calc_* and *σ_u_calc_*.

Table 4 shows the comparative results of the hardening factor *n* obtained from Equation (8) (*n_calc_*) and the value introduced in the FEM simulation (*n_FEM_*). This method showed less accuracy for low values of the hardening factor *n*. It is important to clarify that deviations in the lowest values of the hardening coefficient *n* generate significant deviations in the strain hardening. In addition, high values of the *n* coefficient correspond to materials with low strain hardening, so deviations in these high *n* values generate lower changes in the strain hardening. This is demonstrated in Table 5, which shows the calculated ultimate tensile strength (*σ_u_calc_*) using the *n_calc_* values and Equation (6). Figure 12 shows a comparison of the deviations in the hardening coefficient *n* and the ultimate tensile strength obtained from *n_calc_*. Deviations of the ultimate tensile strength are only high for the lowest value of the hardening coefficient *n*.

To improve these results, the non-linear least squares method of the Matlab^®^ Curve Fitting Tool^TM^ was used to directly obtain the ultimate tensile strength. Thus, the coefficients of Equation (8) were assumed as *A*, *B* and *C* variables, as shown in Equation (9):

(9) $$\;n=\frac{A\sigma_{y}\;}{Slope_{min}-\left(B\sigma_{y}^{2}+C\sigma_{y}\right)}$$

and Equation (9) was introduced in Equation (6):

(10) $$\;ln\left[\frac{\sigma_{u}\;}{\sigma_{y}}\right]=6.142{\left[\frac{Slope_{min}-\left(B\sigma_{y}^{2}+C\sigma_{y}\right)}{A\sigma_{y}}\right]}^{1.326}$$

**Figure 13:**
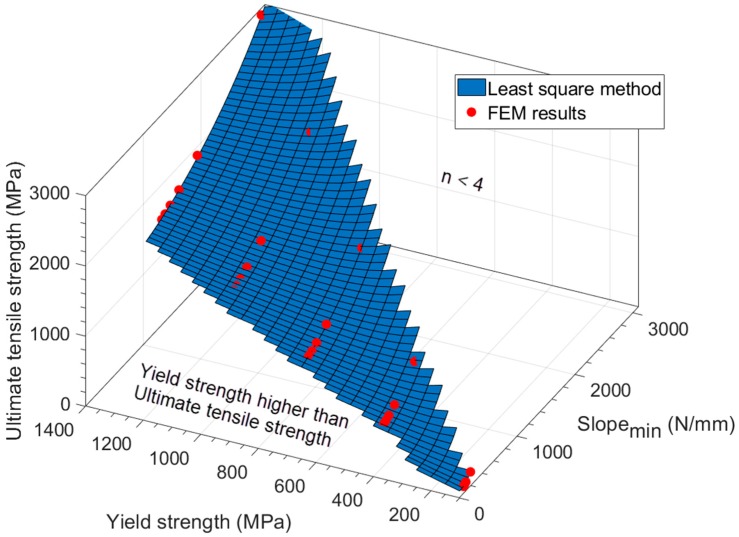
Ultimate tensile strength correlation obtained with Matlab^®^ for the hypothetical materials.

The non-linear least squares method was applied in Equation (10) with FEM data, obtaining the following Equation (11) with *R^2^* = 0.9997 (see Figure 13).

(11) $$\;ln\left[\frac{\sigma_{u}\;}{\sigma_{y}}\right]=6.142{\left[\frac{Slope_{min}-\left(1.973\cdot 10^{-4}\sigma_{y}^{2}+0.377\sigma_{y}\right)}{7.63\sigma_{y}}\right]}^{1.326}$$

The obtained surface in Figure 13 was cut off in two locations: values with hardening coefficient lower than *n* = 4, and values with a yield strength higher than the ultimate tensile strength.

### 3.2. Experimental Tests

**Figure 14:**
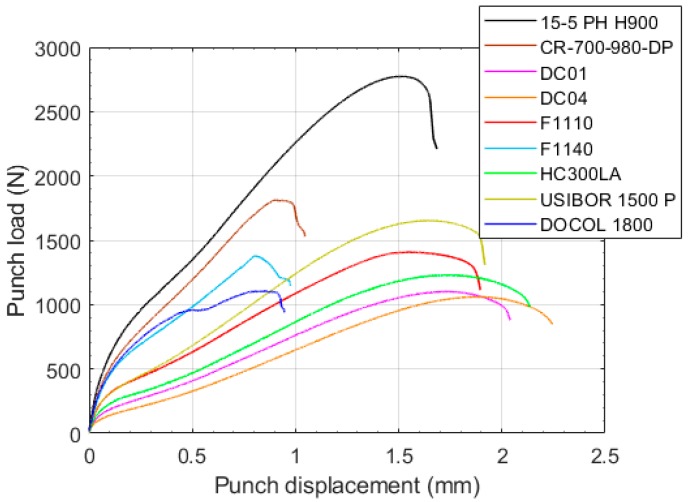
SPT curves of the experimental tests.

Nine steels—DC01, DC04, HC300LA, F1110, F1140, 15-5PH H900, USIBOR 1500 P, CR-700-980-DP, and DOCOL 1800 CR—were tested using standard tensile tests (ASTM E8M) and SPTs to verify the numerical results previously shown. Table 2 shows the mechanical properties for all of the tested materials, and Figure 14 shows the SPT curves obtained from the experimental tests. The geometry and the setup of the SPTs were the same as the ones analyzed in the previous numerical calculations. These experimental data were partially obtained in a previous study [22].

The parameters obtained from these SPT curves are shown in Table 6.

In order to calculate the experimental correlation equation between the ultimate tensile strength and *Slope_min_*, all of the numerical parameters were calculated again with Equation (10). The yield strength used in Equation (10) was the value obtained from the SPT curve via the *Slope_ini_* method (Equation (12)) [22].

(12) $$\;\sigma_{y}=37.437\cdot e^{1.7995\cdot {10\;}^{-4}\cdot Slope_{ini}/t}$$

**Figure 15:**
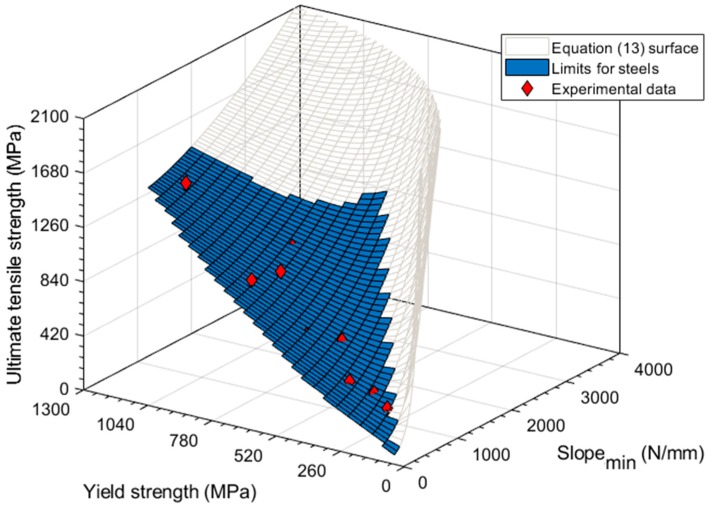
Ultimate tensile strength correlation obtained with Matlab^®^ for the experimental data.

The following Figure 15 and Equation (13) show the results obtained from the Matlab^®^ Curve Fitting Tool^TM^. The coefficient of determination of this regression was *R*^2^ = 0.997.

(13) $$\;ln\left[\frac{\sigma_{u}\;}{\sigma_{y}}\right]=6.142{\left[\frac{Slope_{min}-\left(6.88\times 10^{-4}\sigma_{y}^{2}-0.028\sigma_{y}\right)}{14.68\sigma_{y}}\right]}^{1.326}$$

Table 7 shows the ultimate tensile strength obtained using Equation (13). As commented on previously, the yield strength used for this calculation is derived from the *Slope_ini_* of the SPT curve. The use of the *Slope_ini_* method allows for avoiding the need of the tensile tests to obtain the yield strength of the material.

## 4. Discussion

**Figure 16:**
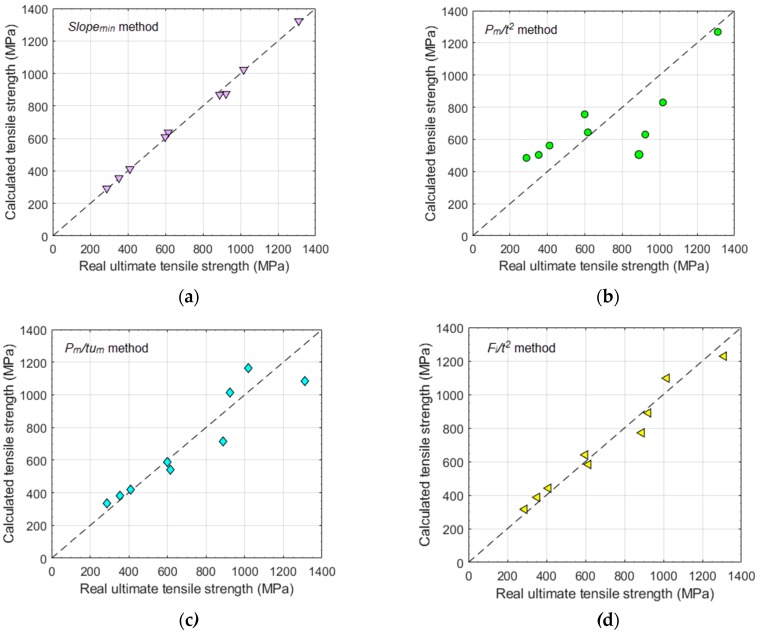
Calculated vs. real ultimate tensile strength of the experimental tests: (**a**) *Slope_min_* method, (**b**) *P_m_*/*t*^2^ method (**c**) *P_m_*/*tu_m_* method (**d**) *F_i_*/*t*^2^ method.

**Figure 17:**
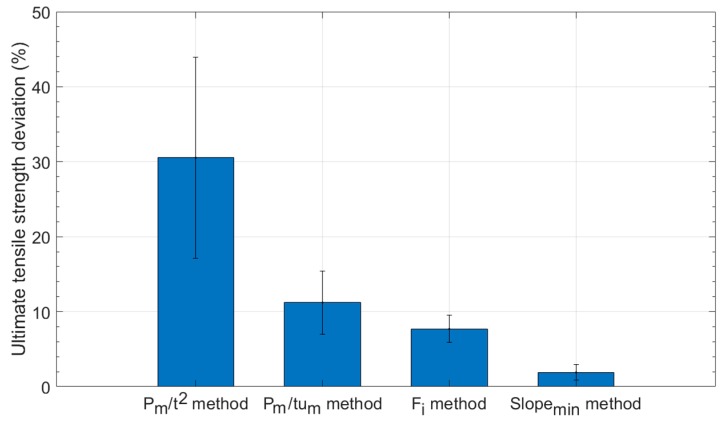
Deviations of the ultimate tensile strength calculation.

In the following Figure 16, a comparison between the ultimate tensile strengths obtained from tensile tests and the ultimate tensile strengths derived from the calculated hardening factors via the *Slope_min_* of the SPT curve is shown in the (a) graph. The rest of the graphs (b to d) shown in Figure 16 represent the results obtained from the most common methods used to correlate the ultimate tensile strength via the *P_m_* parameter (*P_m_/t*^2^ and *P_m_/tu_m_*), and the most recent alternative method via the *F_i_* parameter. Figure 17 shows the deviations between the calculated ultimate tensile strengths from the experimental correlation equations and the ultimate tensile strengths obtained from the tensile tests. The most precise and reliable method was the proposed *Slope_min_* method.

## 5. Conclusions

After these FEM and experimental analyses, the following conclusions were obtained:

1. The dependency of the minimum slope of the SPT curve (*Slope_min_*) with the yield strength and a hardening factor *n* (using the Ramberg–Osgood law) was analyzed and quantified.
2. A novel method to correlate the ultimate tensile strength, via the *Slope_min_*, was obtained showing a good fit (mean deviations of 1.9%). The comparison of this new method with *P_m_* methods (*P_m_/t*^2^ and *P_m_/tu_m_*) and the *F_i_* method showed that the greatest level of fit is reached using the *Slope_min_* method.
3. The *Slope_min_* method combined with the *Slope_ini_* method [22] was shown to be a good methodology for obtaining the yield strength and the ultimate tensile strength using the SPT curve data punch displacements under 0.5 mm. Brittle materials, which show premature failures in the SPT and where a common correlation method fails, could be characterized by these methods (*Slope_ini_* and *Slope_min_*).
4. The experimental tests were done with an analysis of a steel database to guarantee that the obtained correlation equation is applicable to steel alloys with yield strengths lower than 1300 MPa. Taking into consideration that only nine steel alloys were evaluated, a wider set of experimental tests would show more accurate values for the parameters of the equation.

## Figures and Tables

**Table 1:** Plastic properties of the hypothetical materials.

Material	*σ_y_* (MPa)	*n* ^1^	Material	*σ_y_* (MPa)	*n* ^1^
M1.1	100	5	M3.4	750	20
M1.2	100	10	M3.5	750	25
M1.3	100	15	M3.6	750	30
M1.4	100	20	M4.1	1075	5
M1.5	100	25	M4.2	1075	10
M1.6	100	30	M4.3	1075	15
M2.1	425	5	M4.4	1075	20
M2.2	425	10	M4.5	1075	25
M2.3	425	15	M4.6	1075	30
M2.4	425	20	M5.1	1400	5
M2.5	425	25	M5.2	1400	10
M2.6	425	30	M5.3	1400	15
M3.1	750	5	M5.4	1400	20
M3.2	750	10	M5.5	1400	25
M3.3	750	15	M5.6	1400	30

^1^ Ramberg–Osgood parameter.

**Table 2:** Mechanical properties of the experimental materials.

Material	*E* (MPa)	*σ_y_* (MPa)	*σ_u_eng_* (MPa)	*ε_fract_* (mm/mm)
DC04 (1.0338)	203,000	160.0	288.0	0.47
HC300LA (1.0489)	206,000	322.0	411.0	0.31
DC01 (1.0330)	208,000	229.0	353.0	0.35
F1110 (1.0401)	216,430	550.6	615.6	0.19
F1140 (1.1191)	204,910	745.0	922.7	0.10
15-5PH H900 (1.4545)	194,926	1215.0	1310.0	0.16
CR-700-980-DP (1.0997)	207,000	782.0	1017.0	0.11
USIBOR 1500 P	209,000	433.0	599.0	0.18
DOCOL 1800	200,451	807.24	889.07	0.05

**Table 3:** *Slope_min_* of the SPT curves.

Material	*Slope_min_* (N/mm)	Material	*Slope_min_* (N/mm)	Material	*Slope_min_* (N/mm)
M1.1	187.09	M2.5	321.08	M4.3	1151.59
M1.2	107.50	M2.6	303.82	M4.4	1036.66
M1.3	86.30	M3.1	1525.35	M4.5	969.11
M1.4	76.49	M3.2	917.65	M4.6	924.92
M1.5	70.81	M3.3	749.22	M5.1	3015.43
M1.6	67.08	M3.4	669.63	M5.2	1906.80
M2.1	830.73	M3.5	622.76	M5.3	1590.57
M2.2	486.33	M3.6	591.93	M5.4	1440.04
M2.3	391.68	M4.1	2257.54	M5.5	1351.82
M2.4	347.22	M4.2	1394.13	M5.6	1293.41

**Table 4:** Deviations between *n_calc_* and *n_FEM_*.

Material	*n_FEM_*	*n_calc_*	Deviation (%)	Material	*n_FEM_*	*n_calc_*	Deviation (%)
M1.1	5	4.57	8.66	M3.4	20	20.18	0.90
M1.2	10	10.06	0.62	M3.5	25	24.93	0.28
M1.3	15	15.11	0.72	M3.6	30	29.52	1.60
M1.4	20	19.80	1.00	M4.1	5	4.50	9.93
M1.5	25	24.17	3.30	M4.2	10	9.91	0.88
M1.6	30	28.27	5.76	M4.3	15	14.99	0.09
M2.1	5	4.51	9.79	M4.4	20	19.77	1.13
M2.2	10	10.17	1.66	M4.5	25	24.31	2.77
M2.3	15	15.67	4.44	M4.6	30	28.64	4.53
M2.4	20	21.08	5.41	M5.1	5	4.58	8.40
M2.5	25	26.42	5.67	M5.2	10	10.07	0.70
M2.6	30	31.75	5.82	M5.3	15	15.34	2.28
M3.1	5	4.48	10.42	M5.4	20	20.41	2.06
M3.2	10	9.99	0.14	M5.5	25	25.31	1.24
M3.3	15	15.20	1.34	M5.6	30	30.09	0.29

**Table 5:** Deviations between *σ_u_calc_* and *σ_u_FEM_*.

Material	*σ_u_FEM_*	*σ_u_calc_*	Deviation (%)	Material	*σ_u_FEM_*	*σ_u_calc_*	Deviation (%)
M1.1	206.8	226.9	9.72	M3.4	841.9	840.7	0.14
M1.2	133.6	133.3	0.24	M3.5	817.3	817.6	0.03
M1.3	118.4	118.3	0.16	M3.6	802.4	803.5	0.15
M1.4	112.3	112.4	0.15	M4.1	2223.2	2476.9	11.41
M1.5	109.0	109.4	0.39	M4.2	1436.3	1441.2	0.34
M1.6	107.0	107.6	0.55	M4.3	1273.2	1273.5	0.02
M2.1	878.9	977.6	11.23	M4.4	1206.7	1208.8	0.18
M2.2	567.9	564.3	0.62	M4.5	1171.5	1175.3	0.33
M2.3	503.4	498.6	0.94	M4.6	1150.1	1155.0	0.43
M2.4	477.1	473.4	0.78	M5.1	2895.3	3166.9	9.38
M2.5	463.1	460.3	0.60	M5.2	1870.6	1865.6	0.27
M2.6	454.7	452.5	0.49	M5.3	1658.2	1649.9	0.50
M3.1	1551.0	1738.7	12.10	M5.4	1571.5	1566.7	0.31
M3.2	1002.1	1002.6	0.05	M5.5	1525.7	1523.5	0.14
M3.3	888.3	885.7	0.30	M5.6	1497.8	1497.4	0.03

**Table 6:** Experimental SPT parameters.

Materials	*Slope_ini_* (N/mm)	*Slope_min_* (N/mm)	*P_m_* (*N*)	*F_i_* (*N*)	*u_m_* (*mm*)
DC04 (1.0338)	4188.04	416.88	1060.34	416.81	1.869
HC300LA (1.0489)	6016.37	472.06	1228.69	582.82	1.733
DC01 (1.0330)	4889.10	504.12	1100.86	510.99	1.712
F1110 (1.0401)	7419.10	702.63	1407.42	769.95	1.537
F1140 (1.1191)	8200.76	1126.32	1378.22	1176.23	0.805
15-5PH H900 (1.4545)	9782.01	1513.27	2773.47	1622.88	1.513
CR-700-980-DP (1.0997)	8354.51	1516.08	1814.09	1449.30	0.924
USIBOR 1500 P	6861.36	822.61	1652.49	845.65	1.660
DOCOL 1800	6134.37	857.49	1105.43	1019.39	0.915

**Table 7:** Yield strength and ultimate tensile strength obtained by *Slope_ini_* and *Slope_min_* methods.

Materials	*σ_y_* Obtained with *Slope_ini_* Method (MPa)	*σ_ult_* Obtained with *Slope_min_* Method (MPa)
DC04 (1.0338)	169.01	292.86
HC300LA (1.0489)	326.34	412.49
DC01 (1.0330)	217.51	356.68
F1110 (1.0401)	540.66	637.07
F1140 (1.1191)	716.31	874.52
15-5PH H900 (1.4545)	1265.48	1322.19
CR-700-980-DP (1.0997)	757.06	1022.86
USIBOR 1500 P	442.33	609.48
DOCOL 1800	805.22	869.48
